# The Impact of Parental Knowledge and Tanning Attitudes on Sun Protection Practice for Young Children in Germany

**DOI:** 10.3390/ijerph110504768

**Published:** 2014-05-05

**Authors:** Olaf Gefeller, Jiang Li, Wolfgang Uter, Annette B. Pfahlberg

**Affiliations:** Department of Medical Informatics, Biometry and Epidemiology, Friedrich Alexander University of Erlangen-Nuremberg, Erlangen D-91054, Germany; E-Mails: erlking2011@imbe.med.uni-erlangen.de (J.L.); wolfgang.uter@imbe.med.uni-erlangen.de (W.U.); annette.pfahlberg@imbe.med.uni-erlangen.de (A.B.P.)

**Keywords:** skin cancer, tanning, primary prevention, sun protection, ultraviolet radiation

## Abstract

Public health campaigns have improved knowledge on UVR-associated skin cancer risk and increased sun protection awareness. However, tanned skin is still a common beauty ideal. The relationship between knowledge, attitudes and protective behavior is not fully understood yet. A population-based survey was thus performed in the district of Erlangen involving 2,619 parents of 3- to 6-year old children. By means of a self-administered standardized questionnaire parental knowledge about risk factors for skin cancer, their attitudes towards tanning and details of protective measures taken for their children were assessed. The study analyzed specifically the impact of parental tanning attitudes on sun-protective measures for their children while controlling for parental knowledge about skin cancer risk factors. While parental knowledge was significantly (inversely) associated with agreement to the statement “Tanned skin is healthy skin”, this was not the case for “Tanning makes me look better”. Overall, tanning affirmative attitudes were inversely associated with protective measures taken for the children, whereas parental knowledge had a positive impact on sun protection at the beach only. Multivariable analyses provided evidence for an effect of parental attitude on protective behavior independent of parental knowledge. Tanning attitudes and tanned skin as the misguided ideal of beauty need to be addressed in future public health campaigns to enhance the effectiveness of preventive activities in changing sun protective behavior.

## 1. Introduction

Ultraviolet radiation (UVR) plays a decisive role in the pathogenesis of various forms of skin cancer and has been established as their main environmental risk factor [1]. Although the carcinogenic mechanisms triggered by UVR have not been identified in detail, UVR has been classified as a confirmed human carcinogen initiating and promoting skin cancer due to the overwhelming evidence derived from numerous epidemiologic studies [2]. Skin cancer is one of the most common cancer types in fair skinned populations around the world [3]. For several decades the incidences of cutaneous malignant melanoma and non-melanoma skin cancer have been rising rapidly in many countries with fair skinned populations, including Germany and other parts of Europe [4,5,6,7]. According to German cancer registry data, the crude incidence of malignant melanoma amounted to roughly 20 new cases among 100,000 inhabitants in 2006, which means a tripling since the 1980s [8]. Rising levels of individual UVR exposure have been identified as the main driving force of this trend [9,10,11]. 

Public health campaigns for skin cancer prevention have been performed for more than two decades all over the world [12,13,14]. The objective of these campaigns has been to increase knowledge about skin cancer and its risk factors as well as to provide guidance on sun protection [15]. The dissemination of information about the health risks associated with excessive UVR exposure has been the cornerstone of these prevention programs. By improving knowledge and awareness in the population, behavior was expected to change subsequently. In the long term the incidence of skin cancer should then decline. The early Australian experience regarding the effects achieved by educational campaigns was encouraging: significantly increased knowledge, markedly decreased desire to acquire a suntan, and effective changes in protective behavior led to a clear reduction of the risk for getting sunburns, and incidence rates of skin cancer levelled off after decades of steady increase [16]. However, many more recent studies in other areas of the world suggested that such success may be incomplete, *i.e.*, changes in protective behavior had not been fully mirrored the improvement in knowledge [17,18,19,20].

Parents of children have been one of the primary target groups in skin cancer prevention [21,22]. Parents serve as a role model for their children and their educational guidance is essential for behavior-forming of their children, including sun protection. Thus, their attitudes, knowledge concerning UVR exposure and protective behavior have a long-lasting effect on their children [23,24].

While intervention studies designed for educating parents were apparently successful in terms of improving sun protection awareness, their impact on changes in actual protective behavior is not fully satisfactory [17,25]. The barrier to a successful translation of knowledge into effective sun-protective behavior is intertwined with the beauty ideal of tanned skin. Intentional UVR exposure for the purpose of acquiring a tanned appearance is still common in large parts of fair skinned populations [18,26,27,28]. Parental attitudes towards tanning may thus interfere with strict compliance to preventive rules of minimizing UVR exposure. 

In this investigation the mingled relationship of knowledge, tanning attitudes and protective behavior is analyzed based on a large population-based survey. Specifically, the impact of parental tanning attitudes on sun-protective measures for their children is analyzed in a framework allowing statistical control of the confounding effect of parental knowledge. Thereby, the question whether there is an independent effect of tanning attitudes on behavior or whether effects of tanning attitudes can be explained by differences in knowledge levels can be addressed. 

## 2. Experimental Section

### 2.1. Participants

Details of the design, recruitment strategy and conduct of the cross-sectional Erlangen Kindergarten (ErlKing) study have been published elsewhere [29]. In brief, we enrolled participants from the population in the Northern Bavarian district of Erlangen, which comprises the city of Erlangen and its surrounding rural county. Using official administrative information we selected 59 of the 118 kindergartens in the study region. This selection was, however, not the result of random sampling as we excluded those with a low number of children for logistical reasons. Overall, 4,146 questionnaires were distributed to parents via the kindergarten teachers during the winter 2001/2002. If a family had two or more children attending the kindergarten, only the data of the oldest child were included in the analysis. A total of 2,682 questionnaires were returned, yielding a response rate of 64.7%. Data of 63 children were excluded from the final analysis as the children’s age fell outside the pre-specified range of 3 to 6 years. Of the final 2,619 questionnaires, 87.7% were completed by the mother alone, 5.4% by the father alone, 6.2% by both parents, and the small remainder by other family members. The study was approved by the local ethics committee at the University of Erlangen-Nuremberg. 

### 2.2. Questionnaire

The self-administered standardized questionnaire contained demographic characteristics, such as the child’s age and gender, maternal and paternal age, and photosensitivity data, such as the child’s hair and iris color. The majority of items addressed parental attitudes, knowledge about skin cancer risk factors and sun protective behavior for the children. In more detail:

*Attitude*: The parental attitude towards tanning was measured by the level of agreement to the two items: (i) “Tanned skin is healthy skin.” and (ii) “Tanning makes me look better.” Parents could select their answer from a 4-point Likert scale (“totally agree”, “agree partially”, “tend to disagree”, “totally disagree”). 

*Knowledge*: Nine exposures were listed in the questionnaire and parents had to judge whether or not these were risk factors for skin cancer. The nine exposures included six true risk factors (such as “number of nevi”, “frequency of sunburns during childhood”, “fair skin, fair hair”, “intermittent intensive sun exposure”, “sunbathing during life”, “chronic sun exposure”). As additional factors three exposures (“presence of allergies”, “increasing air pollution”, and “unhealthy diet”) were also included in the list to check whether parents were able to distinguish between true risk factors and those exposures attracting public concern, but not having been identified as skin cancer risk factors. The pattern of answers was summarized by a sum score developed in an earlier study using the same items for assessing knowledge about risk factors for skin cancer [30]. For the analysis the score that was further classified into three categories (“low”, “medium”, “high”).

*Protective behavior*: Parents were asked regarding typical instructions given to their children when these played outside on a summer day in two different settings, namely, in the garden or the playground as an everyday setting and during beach holidays. Four aspects relevant for sun protection were ascertained: (i) type of clothes worn; (ii) frequency of staying in the shade; (iii) wearing a sunhat; and (iv) frequency of using sunscreens. The item “wearing a sunhat” was dichotomously assessed as “yes” or “no”. In contrast, the other three items were originally assessed with several categories and later dichotomized for the analysis as follows: clothing into “unprotected” (naked or swimsuit) *vs.* “protected” (T-shirt or long-sleeved clothes); shade into “unprotected” (rarely or occasionally) *vs.* “protected” (mostly or always); sunscreen into “unprotected” (none or rarely) *vs.* “protected” (every 2 to 3 hours). The same dichotomizations have been used in a previous ErlKing analysis [31].

### 2.3. Statistical Analysis

Data analysis was performed using SAS 9.3 (SAS Institute Inc., Cary, NC, USA). The crude (bivariate) associations between parental attitude, knowledge, and measures of sun protection were assessed using the Mantel-Haenszel χ^2^ test. 

Log-linear models were then analyzed to identify statistically significant interactions between knowledge, attitude, and sun protective measures in a multifactorial framework including as confounding factors also iris color and hair color, respectively. In total, 32 log-linear models were constructed by the use of different variable combinations. All models initially included all main effects as well as all first- and second-order interactions. After a backward elimination procedure preserving model hierarchy, the significant items stayed in final models. 

Finally, logistic regression analyses were used to quantify the impact of parental attitude and knowledge on sun protection practice in separate models for the four protective measures. For this purpose, both attitude items were reclassified into dichotomous indicators (“agree *vs.* disagree”) and the knowledge score was also dichotomized (“low or medium” *vs.* “high”). In a next step, these dichotomized parental attitudes and knowledge indicators were used to define a new explanatory factor with four levels by cross-classifying the two dichotomous indicators. The combined 4-level variable enables a better statistical separation of the interacting effects of knowledge and attitude. As we consider two different tanning attitudes, four protective measures and two environmental settings, sixteen logistic models had to be analyzed. Children’s iris color as an indicator of photosensitivity and their age were included additionally in all models as potential confounders. The impact of explanatory factors was quantified by adjusted odds ratios (ORs) and their 95% confidence intervals (CIs) based on the profile likelihood method. *P* values of <0.05 from two-sided statistical tests were considered statistically significant in all analyses.

## 3. Results

Forty-nine per cent of the children in the survey were girls. The age of mothers ranged from 18 to 51, the age of fathers ranged from 21 to 69, the average being 34.3 ± 4.6 and 36.9 ± 5.3 (mean ± standard deviation) years, respectively. Hair color of the children was fair in 65%, irises blue or green in 68%. There were virtually no differences between genders regarding the distributions of photosensitivity attributes.

As shown in Table 1, parental knowledge was significantly associated with the attitude towards “Tanned skin is healthy skin”. Parents agreeing to “Tanned skin is healthy skin” had a lower level of knowledge. On the other hand, there was no significant relationship between knowledge and agreement to the statement “Tanning makes me look better”. 

Table 1 furthermore shows the distribution of the four protective measures in both garden and beach settings according to the levels of agreement to the two attitude statements. It is evident that agreeing on “Tanned skin is healthy skin” is significantly (inversely) associated with all four protective measures, in both garden setting and beach setting, respectively. Such a relationship was also found between the parental attitude towards “Tanning makes me looks better” and most protective measures for children, except regarding the use of sunscreens and wearing a sunhat in the beach setting.

**Figure 1:**
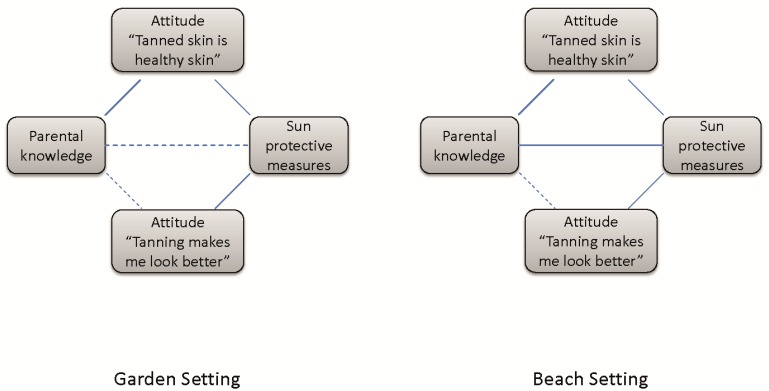
The relationship between parental knowledge, attitudes towards tanning and sun protective measures—visualization of results from log-linear modeling (see text).

Results from log-linear modeling revealed that no significant interaction existed between knowledge and all four protective measures in the garden setting and only for two items (clothing, use of sunscreens) in the beach setting. The results from the bivariate analyses (Table 1) regarding the association between knowledge and attitudes towards tanning as well as between attitudes and protective measures taken for the children were corroborated in these multivariable analyses; no significant interactions between more than two factors were identified. The results of all 32 log-linear models were consistent irrespective of which confounding variables were additionally incorporated into the models. The relationships between variables are illustrated in Figure 1, where the dashed lines visualize non-significant associations and the solid lines emphasize the significant associations observed.

**Table 1:** The bivariate association of parental attitudes towards tanning with parental knowledge and sun protective measures for their children in the ErlKing study (n = 2,619).

	Sample Size (%)	Tanned skin is healthy skin					Tanning makes me look better				
		Totally Agree	Partially Agree	Partially Disagree	Totally Disagree	*p*	Totally Agree	Partially Agree	Partially Disagree	Totally Disagree	*p*
Skin cancer risk factors knowledge											
High	1068 (40.8)	3 (6.1)	250 (32.9)	356 (41.0)	440 (49.7)	<0.01	203 (36.2)	628 (44.1)	130 (41.7)	99 (34.7)	0.78
Medium	967 (36.9)	25 (51.0)	281 (37.0)	334 (38.5)	308 (34.8)		214 (38.1)	508 (35.6)	114 (36.5)	121 (42.5)	
Low	584 (22.3)	21 (42.9)	229 (30.1)	178 (20.5)	138 (15.6)		144 (25.7)	289 (20.3)	68 (21.8)	65 (22.8)	
Garden setting											
Clothing											
Unprotected	371 (14.2)	8 (16.7)	123 (16.3)	124 (14.3)	105 (11.9)	0.01	87 (15.6)	212 (14.9)	36 (11.6)	30 (10.5)	0.02
Protected	2239 (85.8)	40 (83.3)	633 (83.7)	743 (85.7)	779 (88.1)		470 (84.4)	1211 (85.1)	274 (88.4)	255 (89.5)	
Shade											
Unprotected	1154 (44.3)	22 (44.9)	387 (51.2)	391 (45.3)	333 (37.8)	<0.01	282 (50.5)	632 (44.7)	120 (38.5)	106 (37.2)	<0.01
Protected	1451 (55.7)	27 (55.1)	369 (48.8)	472 (54.7)	549 (62.2)		277 (49.5)	781 (55.3)	192 (61.5)	179 (62.8)	
Sunhat											
No	664 (25.7)	13 (27.1)	229 (30.6)	221 (25.8)	189 (21.7)	<0.01	177 (31.9)	337 (23.9)	79 (25.7)	61 (21.9)	<0.01
Yes	1916 (74.3)	35 (72.9)	520 (69.4)	635 (74.2)	682 (78.3)		377 (68.1)	1070 (76.1)	228 (74.3)	217 (78.1)	
Sunscreen											
Unprotected	1834 (70.6)	41 (85.4)	560 (74.3)	593 (68.9)	600 (68.3)	<0.01	399 (71.6)	1011 (71.6)	214 (69.3)	184 (65.0)	0.04
Protected	762 (29.4)	7 (15.6)	194 (25.7)	268 (31.1)	279 (31.7)		158 (28.4)	402 (28.4)	95 (30.7)	99 (35.0)	
	Beach setting										
Clothing											
Unprotected	1632 (67.8)	33 (80.5)	505 (74.5)	561 (68.9)	502 (60.6)	<0.01	393 (74.1)	889 (67.7)	178 (62.7)	154 (60.9)	
Protected	774 (32.2)	8 (19.5)	173 (25.5)	253 (31.1)	326 (39.4)		137 (25.9)	425 (32.3)	106 (37.3)	99 (39.1)	<0.01
Shade											
Unprotected	752 (31.4)	10 (24.4)	248 (36.6)	260 (32.0)	220 (26.8)	<0.01	183 (34.8)	434 (33.0)	66 (23.7)	61 (24.3)	
Protected	1644 (68.6)	31 (75.6)	430 (63.4)	552 (68.0)	600 (73.2)		343 (65.2)	881 (67.0)	213 (76.3)	190 (75.7)	<0.01
Sunhat											
No	217 (9.1)	5 (12.5)	82 (12.2)	69 (8.6)	57 (7.0)	<0.01	47 (8.9)	123 (9.5)	23 (8.2)	20 (8.2)	0.60
Yes	2164 (90.9)	35 (87.5)	590 (87.8)	737 (91.4)	760 (93.0)		479 (91.1)	1179 (90.5)	259 (91.8)	225 (91.8)	
Sunscreen											
Unprotected	497 (20.7)	15 (36.6)	160 (23.7)	163 (20.0)	154 (18.6)	<0.01	117 (22.3)	265 (20.1)	56 (19.7)	53 (21.0)	0.58
Protected	1905 (79.3)	26 (63.4)	516 (76.3)	651 (80.0)	673 (81.4)		407 (77.7)	1052 (79.9)	228 (80.3)	199 (79.0)	

**Figure 2:**
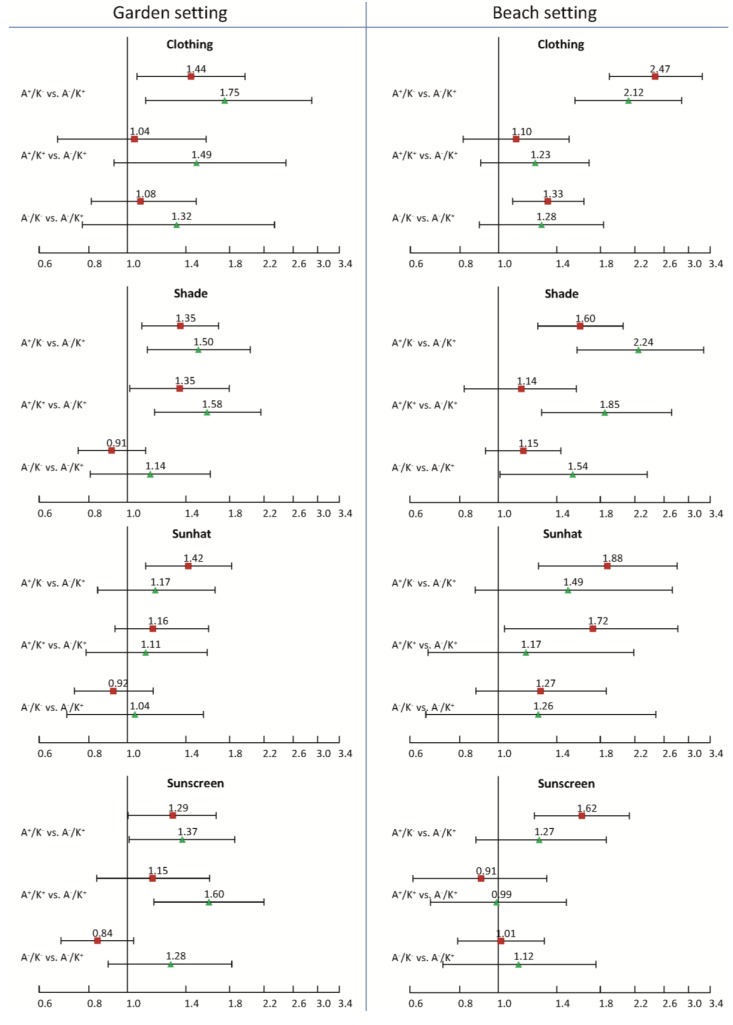
The combined impact of parental knowledge and two (separate) attitudes towards tanning on sun protection practice (four behavioral items) for their children.

Figure 2 illustrates the pattern of results derived from logistic regression analyses. The impact of the attitude-knowledge combination on the four protective measures for children in two environmental settings, namely the garden and the beach environment, are compared across the different situations. The ORs shown in the figure describe the risk for the children being unprotected for some category of the attitude-knowledge combination relative to the same risk in the reference category. The reference category in this analysis is formed by children with parents with a high level of knowledge (K+) and a critical attitude towards tanning (A‒). In most situations the highest ORs were found in the subgroup of parents with a low level of knowledge and a positive attitude towards tanning. For example, the risk of not wearing appropriate protective clothes at the beach was more than doubled in the subgroups with a low level of knowledge (K−) and agreeing to the statement “Tanned skin is healthy skin” (OR = 2.47, 95% CI: 1.89–3.25) and “Tanning makes me look better” (OR = 2.12, 95% CI: 1.55–2.88), respectively. The comparison of results for the two different attitudes towards tanning gave a mixed picture. In the garden setting higher ORs—for all comparable situations except in the sunhat case—were observed for the statement “Tanning makes me look better” pointing to a stronger effect of this parental attitude on the intensity of sun protection for the children. This pattern could not be confirmed for the beach setting, where different results emerged for different protective measures.

In Figure 2 the explanatory factors defined by the attitude-knowledge combination have the following four levels:

A+/K−: Attitude: agree combined with Knowledge: low or medium;
A−/K−: Attitude: disagree combined with Knowledge: low or medium;
A+/K+: Attitude: agree combined with Knowledge: high;
A−/K+: Attitude: disagree combined with Knowledge: high (the reference group).

The adjusted ORs for the attitude “Tanned skin is health skin” are represented by red squares (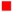) and regarding “Tanning makes me look better” by green triangles (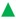). The widths of the accompanying 95% CIs are shown as solid horizontal bars. The ORs describe the adjusted odds for children being unprotected for A+/K−, A−/K−, and A+/K+, respectively, relative to the reference category A−/K+. 

The results also provided evidence for an effect of parental attitude on protective behavior independent of parental knowledge. When looking at the ORs for the subgroup (A+/K+), which can always be found in the middle of the single subfigures in Figure 2, the impact of a positive attitude towards tanning can be evaluated among those with a high level of knowledge. In the garden and the beach setting a strong increase of the risk being unprotected could be identified in several cases. For example, parents who agreed on “Tanning makes me look better” instructed their children significantly less often to stay protected in the shade. The ORs for being unprotected amounted to 1.85 (95% CI: 1.28–2.71) in the beach setting and to 1.58 (95% CI: 1.16–2.15) in the garden setting, respectively. 

## 4. Discussion

The interplay between parental knowledge, attitudes and beliefs impacts behavioral guidance on sun protection given to children. If only knowledge would determine protective behavior, skin cancer prevention would be simple. Empirical evidence from a variety of studies has shown that it is not that simple [17,32,33,34]. Obviously, health beliefs and tanning attitudes play a role in this mingled situation and can jeopardize protective activities of skin cancer prevention programs. 

Previous studies have used a diversity of approaches and specific measures to capture parental knowledge, (tanning) attitudes and related psychosocial constructs. A recent review by Tripp *et al.* [35] criticized the lack of a theoretic foundation and standardized operational definitions of these constructs employed in the 57 studies covered by the review. Our approach, developed in the early 90ies for previous surveys [30,36,37,38], used a sum score based on nine items to measure knowledge about skin cancer risk factors and a two-dimensional assessment of tanning attitudes which has not been summarized further. The two dimensions reflect different aspects of the perception of tanned skin. One dimension refers to the health perception of a tanned appearance (“Tanned skin is healthy skin”), the other refers to the beauty aspect of tanned skin (“Tanning makes me look better”). In our data, knowledge about skin cancer risk factors was associated with the health aspect, whereas this was not the case regarding the beauty aspect. It could be speculated that in people aiming at being attractive by means of tanning, this desire overrides their intellectual knowledge about the risk of UVR exposure [39,40]. This is accordance with the finding of a study by Knight *et al.* among US college students [41], in which despite adequate knowledge of the adverse effects of UVR exposure more than 90% of responders freely and frequently used tanning lamps for beauty reasons. This underlines that a mere enhancement of knowledge is not sufficient to change the attitude towards tanning, especially when tanned skin is regarded as an ideal of beauty. As a consequence some modern intervention programs adopted a new strategy by linking UVR exposure to deterioration of appearance, *i.e.*, skin aging, to alter tanning attitudes and to enhance sun protection intentions and behavior. In a recent review by Williams *et al.* [42] the authors conclude that this approach is promising and may have a role in health promotion. 

Our analysis addressed—for the first time in studies on this topic—the joint (and separate) effect(s) of parental knowledge and tanning attitudes on the implementation of protective measures into educational practice. A better separation of effects in this complex situation was possible due to our modeling approach incorporating potential interactions. Not surprisingly, parents who had a low level of knowledge and valued tanned skin as either attractive or even healthy protected their children less sufficiently than parents with a high level of knowledge and a critical attitude towards tanning. The comparison of risk estimates for other levels of the attitude-knowledge combinations in the models indicated, however, that the attitude focusing on the beauty aspect of tanning had an impact on protective behavior that is independent from the level of parental knowledge. Consequently, even parents with a high level of knowledge about skin cancer risk factors do not protect their children adequately given they value tanned skin as being beautiful.

Our results have also corroborated findings from previous studies that parental attitudes towards tanning are associated with protective measures employed for young children. Some inconsistencies in this overall pattern found in our data can be explained easily. For instance, agreement to “Tanning makes me look better” was not (inversely) associated with wearing a sunhat, likely because this clothing item is not primarily seen as a means of sun protection, but as a fashion element. Furthermore, this attitude was also not (inversely) associated with the use of sunscreens on the beach. In the beach setting, intentional sun exposure to acquire a tan can often be observed. As mentioned by Autier [43], sunscreens provide a (false) sense of security in terms of protection against skin cancer, and encourage a prolonged stay in the sun. In contrast, sunscreen use during daily life activities (the “garden setting”) is a much clearer expression of a deliberate protection against UV exposure without the aim of acquiring a tan. In this garden setting we found a significant (inverse) association between the use of sunscreens and the above attitude towards tanning.

Some limitations should be considered when interpreting our data. Firstly, the general problem of self-selection in population-based surveys and the problematic validity of self-administered questionnaires could potentially interfere with the validity of the results. It can never be ruled out that the group of non-responders, roughly one third of the households with young children in our target population, behave differently than the responding parents. Although a very similar version of our self-administered questionnaire had been used in two earlier surveys in another German city [30,36] and was extensively pretested during the preparation of the current study in Northern Bavaria, we cannot be sure that all responders understood the items as intended. Secondly, validation of instruments employed to assess knowledge, attitudes and behavior has been limited to an internal validation in this and earlier surveys from our group. Thirdly, there are probably more complex relationships between attitude, knowledge, behavior and other factors which we could not incorporate into the analyses of our study. For example, socioeconomic factors [44] and family history of skin cancer [45] were not included in the questionnaire of our survey, although they may act also as additional determinants of relevant behavior. We omitted questions ascertaining this information because of concerns regarding a deterioration of the response rate.

## 5. Conclusions

We addressed the complex relationship between parental knowledge, attitude towards tanning and protective behavior for young children in a large population-based survey. We could demonstrate that even parents with a high level of knowledge about skin cancer risk factors do not protect their children adequately given they have an uncritical attitude towards tanning. Future public health campaigns should thus no longer focus on the mere improvement of knowledge. Targeting tanned skin as a misguided ideal of beauty and thereby causing a change of attitudes towards tanning seems to be better suited to improve the current situation of skin cancer prevention.
